# Risk markers for fatal and non-fatal prescription drug overdose: a meta-analysis

**DOI:** 10.1186/s40621-017-0118-7

**Published:** 2017-08-07

**Authors:** Joanne E. Brady, Rebecca Giglio, Katherine M. Keyes, Charles DiMaggio, Guohua Li

**Affiliations:** 1 Department of Epidemiology, Columbia University Mailman School of Public Health, New York, NY USA; 2 Center for Injury Epidemiology and Prevention, Columbia University, New York, NY USA; 3 Department of Surgery, Division of Trauma, New York University, New York, NY USA; 4 Department of Anesthesiology, Columbia University College of Physicians and Surgeons, New York, NY USA

**Keywords:** Accidents, Analgesics, Opioid/toxicity, Drug overdose, Mortality, Prescription drugs/toxicity, Prevalence, Public health, Risk factors, Substance-related disorder

## Abstract

**Background:**

Drug overdose is a public health crisis in the United States, due in part to the unintended consequences of increases in prescribing of opioid analgesics. Many clinicians evaluate risk markers for opioid-related harms when prescribing opioids for chronic pain; however, more data on predictive risk markers are needed. Risk markers are attributes (modifiable and non-modifiable) that are associated with increased probability of an outcome. This review aims to identify risk markers associated with fatal and non-fatal prescription drug overdose by synthesizing findings in the existing peer-reviewed and grey literature. Eligible cohort, case-control, cross-sectional, and case-cohort studies were reviewed and data were extracted for qualitative and quantitative synthesis.

**Findings:**

Summary odds ratios (SOR) were estimated from 29 studies for six risk markers: sex, age, race, psychiatric disorders, substance use disorder (SUD), and urban/rural residence. Heterogeneity was assessed and effect estimates were stratified by study characteristics. Of the six risk markers identified, SUD had the strongest association with drug overdose death (SOR = 5.24, 95% confidence interval (CI) = 3.53 - 7.76), followed by psychiatric disorders (SOR = 3.94, 95% CI = 3.09 - 5.01), white race (SOR = 2.28, 95% CI = 1.93 - 2.70), the 35-44 year age group relative to the 25-34 year reference group (SOR = 1.52, 95% CI = 1.31 - 1.76), and male sex (SOR = 1.33, 95% CI = 1.17 - 1.51).

**Conclusions:**

This review highlights fatal and non-fatal prescription drug risk markers most frequently assessed in peer-reviewed and grey literature. There is a need to better understand modifiable risk markers and underlying reasons for drug misuse in order to inform interventions that may prevent future drug overdoses.

## Review

Unintentional drug poisoning (overdose) is defined by the Centers for Disease Control and Prevention (CDC) as accidental harm caused by the ingestion, inhalation, injection or absorption of a substance that is not intended to cause harm (Centers for Disease Control and Prevention 2015). Overdose deaths have been increasing for the past 20 years (Warner et al. 2011; Centers for Disease Control and Prevention 2012; Rudd 2016); prescription drug overdose (PDO) deaths have played a considerable role in the drug overdose epidemic (Centers for Disease Control and Prevention 2012; Warner et al. 2009; Okie 2010). Increases in PDO deaths have coincided with an increase in nonmedical use of prescription drugs and prescription drug-related morbidity (Centers for Disease Control and Prevention 2012; Okie 2010; Centers for Disease Control and Prevention 2011; Dhalla et al. 2009; Coben et al. 2010; Cai et al. 2010). The rise in overdose is largely attributable to greater therapeutic and nonmedical prescription drug use and abuse, specifically use and abuse of opioid analgesics (Centers for Disease Control and Prevention 2015; Centers for Disease Control Prevention 2005; Centers for Disease Control Prevention 2011; Mueller et al. 2006; Wunsch et al. 2009; Centers for Disease Control Prevention 2012). These increases have led to numerous regulatory changes in many states, as well as a new prescribing guideline to help providers evaluate risk markers for opioid-related harms when prescribing opioids for chronic pain (Dowell et al. 2016).

Risk markers are attributes that are associated with increased probability of an outcome, and may or may not be modifiable or causal factors (Burt 2001). PDO risk markers may be important for informing public health interventions (Keyes et al. 2014; Diez Roux 2004). However, studies of PDO have not consistently identified the same risk markers or the same effects (Bohnert et al. 2012; Bohnert et al. 2011; Dunn et al. 2010; Havens et al. 2011a; Cochella and Bateman 2011; McKenzie and McFarland 2007; Silva et al. 2013) and there has not been a systematic evaluation of risk markers associated with PDO. Extant reviews and studies of PDO and drug-related mortality have been narrative or have focused on trends over time, but have not systematically evaluated the evidence in support of various risk markers (Warner et al. 2011; Warner et al. 2009; Centers for Disease Control Prevention 2005; Buckley and McManus 2004; Crombie and McLoone 1998; Gilchrist et al. 2012; Bateman et al. 2003; Degenhardt et al. 2001; Fernandez et al. 2006; Fischer et al. 2006; Green et al. 2011; Hall et al. 1999; Lynskey and Hall 1998; Paulozzi and Xi 2008; Paulozzi et al. 2006; Rosca et al. 2012; Romelsjo et al. 2010; Roxburgh et al. 2011; Shah et al. 2005; Shah et al. 2012; Williamson et al. 1997; Wong et al. 2010; Harlow 1991; Lloyd and McElwee 2011; Paulozzi and Stier 2010; Paulozzi et al. 2012).

This review aims to provide a comprehensive and quantitative evaluation of the existing literature concerning the most frequently examined risk markers associated with PDO. Six commonly examined risk markers were identified for further investigation: sex, age, race, comorbid psychiatric disorder, comorbid substance use disorder (SUD) and urban/rural area of residence. Understanding risk markers may help identify modifiable factors which may be intervened upon, and aid in the identification of individuals at greatest risk for PDO. This review examines characteristics of previous research studies, in order to understand differences in their findings, identify salient gaps in the PDO literature and ultimately to help inform policy and guide decision-making regarding preventive interventions to curb PDO.

## Methods

Reporting in this meta-analysis followed standard methodology, adhering to the procedural and reporting recommendations for conducting meta-analyses outlined in the PRISMA statement and MOOSE guidelines (Moher et al. 2009; Stroup et al. 2000).

### Eligibility

This review contains two components: 1) a systematic descriptive review, and 2) a systematic quantitative meta-analysis. To be included, a study must have been published in the English language from January 1, 1990 to December 1, 2016. Included studies used a standard epidemiologic study design (e.g., cohort, case-control, cross-sectional, case-cohort study or survey) from which effect measures could be extracted. Publications reporting on case-series (e.g., medical examiner data with no non-events and not including rates, comparative case-series, letters, editorials commentaries, opinion pieces), case reports, reviews, time-series or trend studies were excluded. If two studies examined the same outcome in the same individuals during the same time period, but examined a different control series, only the study published first was included (Lanier et al. 2012; Johnson et al. 2012; Cheng et al. 2013).

In this review, prescription drugs are defined as any drug requiring a prescription from a licensed healthcare provider, including controlled and non-controlled substances. Studies that focus on illegal drugs, excluding prescription drugs, or that give no indication of prescription drug use were excluded. As this review examines factors associated with accidental (fatal and non-fatal) PDO, studies that exclusively examine suicide or self-poisoning were excluded. Further, studies that did not consider risk markers for overdose were excluded, as were studies focusing on: infants or children under 12; overdose after drug treatment; recurrent overdose; or the effects of an intervention or policy.

### Databases, search strategy and criteria

Relevant literature was identified through electronic searches of databases: Medline OVID (1946–present), Cochrane Library (1960–present), Cumulative Index to Nursing and Allied Health Literature (1981–present), PsycInfo (1967–present), Scopus (1996–present) and ISI Web of Knowledge (1968–present). Relevant literature examined included peer-reviewed, published papers, abstracts and papers presented at scientific conferences, as well as “grey literature”. Grey literature was identified through manual review of relevant reference lists. Electronic databases were searched using Medical Subject Heading keywords for indexed databases (Medline and PsycInfo) and keywords for indexed and non-indexed databases. A medical librarian was consulted to review the databases searched, search terms and search strategy (Appendix 1).

### Study selection

After electronic search, duplicate citations were removed. Titles of references were reviewed for relevancy to ensure the article examined PDO. The abstracts of potentially relevant titles were reviewed to further assess a study’s eligibility for inclusion. If the study abstract was considered eligible, the full text of the study was retrieved and evaluated to determine if the study was eligible for inclusion. Two reviewers independently reviewed the full text of studies identified for inclusion. In cases of disagreement, the study was discussed until consensus was reached.

### Data extraction

Information about the characteristics of each study was extracted, and data were extracted to identify the most commonly studied risk markers, and to calculate the unadjusted odds ratio of PDO. This was done in two steps: 1) information on risk markers for PDO was collected from each study, and 2) a list was generated to compare which risk markers for PDO were most frequently examined. Risk marker information was categorized into common domains, and risk markers were consolidated where possible (not shown). If a paper was deemed eligible for inclusion, but data on the number of individuals with the outcome or the risk marker of interest were not extractable, the corresponding author of the paper was contacted for additional information. Articles meeting these criteria were selected for qualitative review and characteristics of these studies were reviewed. Risk markers (sex, age, race, comorbid psychiatric disorder, comorbid SUD and urban/rural of the area of residence) were selected for the quantitative analysis when five or more studies examined the same risk marker.

### Quality assessment

After full-text review, the quality of studies eligible for inclusion was evaluated. Quality of relevant studies identified through automated search and hand search of references was evaluated using the Newcastle–Ottawa Quality Assessment Scale (Wells et al. 2011). Quality assessments of cross-sectional studies were evaluated using the modified Newcastle–Ottawa Quality Assessment Scale (Herzog et al. 2013).

### Data synthesis and analysis

The unadjusted odds ratio measuring the association of each risk marker with PDO was estimated for each study. Forest plots were created to show the distribution of effect estimates across studies for each risk marker studied. Heterogeneity was assessed using two statistics – Cochran’s Q test statistic and its corresponding *p*-value, and I^2^ statistics. The Q statistic tests the null hypothesis that each study evaluates the same effect, whereas the I^2^ indicates the proportion of total variation across studies that is due to unexplained heterogeneity (Higgins and Thompson 2002). For the Q statistic, a *p*-value of ≤0.05 was considered statistically significant and the effect estimates from the studies were considered heterogeneous. An I^2^ above 0.5 is considered heterogeneous (Higgins and Thompson 2002). If a heterogeneous result is found, summary effect estimates from a random effects models should be considered (Riley et al. 2011). For comparison purposes and as a test of sensitivity of the results to model choice, the results of both fixed and random effects model are presented in all forest plots.

When the number of studies permitted, sources of heterogeneity were investigated by stratification analysis according to study design, study quality assessment score, fatal vs combined fatal and nonfatal overdose outcomes, and whether the study outcome was any overdose, medication overdose, PDO or prescription opioid overdose. Heterogeneity was evaluated for each stratified analysis. “One study removed” sensitivity analyses were conducted to assess the robustness of the summary odds ratio. Publication bias was assessed with funnel plots (not shown) and Rosenthal’s fail-safe N (Persaud 1996). Analyses were conducted in Microsoft Excel 2010 (Microsoft Corporation, Redmond, Washington), Comprehensive Meta-Analysis version 2 (Biostat Inc., Englewood, New Jersey) and SAS version 9.4 (Statistical Analysis Software, Cary, North Carolina).

## Results

**Fig. 1:**
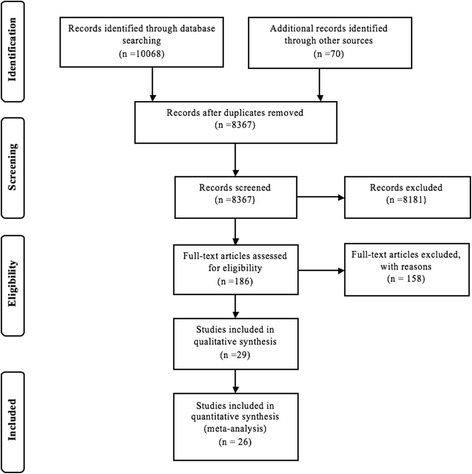
Flow diagram of the identification, review and selection of included prescription drug overdose meta-analysis articles. Footnote: Adapted From: (Moher et al. 2009)

Electronic database searching generated 10,068 references of potential relevance. An additional 70 references were identified through manual review of the references of the studies that underwent quality assessment. From the potentially relevant references, 1771 duplicate references were removed, leaving a total of 8367 records to be screened by title, study design and abstract content. After title review and exclusion of commentaries, and case reports, 186 references remained. The full-texts of these references were acquired. After further review, 29 studies were deemed eligible for the qualitative and quantitative synthesis (Fig. 1). These 29 studies were evaluated according to their study design using the Newcastle–Ottawa Quality Assessment Scale (Tables 1, 2, 3). In this version of the assessment scale, the best possible assessment scale score varies by study design, where the highest possible score for case-control and cross-sectional studies is a 10 and the highest possible score for cohort studies is a 9. A higher score denotes a better quality study. Overall, the case-control and cohort studies were of high quality, with mean assessment scale scores of 9.0/10 and 8.1/9 for case-control and cohort studies respectively. The cross-sectional studies had a mean scale score of 5.4/10.

**Table 1:** Modified Newcastle-Ottawa quality assessment scale ratings for the 10 case-control or case-cohort studies included

	Selection (score)				Comparability (score)	Exposure (score)			Total score
	Case definition	Representative of cases	Selections of controls	Definition of controls	Control for important covariates	Ascertainment of exposure	Same method of ascertainment for participants	Nonresponse rate	Out of 10 points
Bohnert et al. 2011	1	1	2	1	2	1	1	1	10 (high)
Brady et al. 2015	1	1	2	1	2	1	1	1	10 (high)
Cerdá et al. 2013	1	1	1	1	2	1	1	1	9 (high)
Dilokthornsakul et al. 2016	1	1	2	1	2	1	1	1	10 (high)
Gomes et al. 2011	1	1	2	1	2	1	1	1	10 (high)
Lanier et al. 2012	1	1	1	1	2	0	0	0	6 (low)
Paulozzi et al. 2012	1	1	2	1	1	1	1	1	8 (high)
Peirce et al. 2012	1	1	2	1	0	1	1	1	8 (high)
Whitmire and Adams 2010	0	1	2	1	2	1	1	1	10 (high)
Zedler et al. 2014	1	1	2	1	2	1	1	1	10 (high)
								mean	9.0

**Table 2:** Newcastle-Ottawa quality assessment scale ratings for the 7 cohort studies included

	Selection				Comparability	Outcome			Total score
	Representative of exposed cohort	Selections of non exposed cohort	Assessment of exposure	Absence of outcome at start of study	Comparability	Assessment of outcome	Follow-up period (≥ 6 months)	Adequacy of follow-up	Out of 9 points
Bauer et al. 2016	1	1	1	1	2	1	1	0	8 (high)
Bohnert et al. 2012	1	1	1	1	2	1	1	0	8 (high)
Caudarella et al. 2016	1	1	1	1	2	1	1	1	9 (high)
Dunn et al. 2010	1	1	1	1	2	1	1	0	8 (high)
Hartung et al. 2007	1	1	1	1	2	1	1	0	8 (high)
Seal et al. 2012	1	1	1	1	2	1	1	0	8 (high)
Turner and Liang 2015	1	1	1	1	2	1	1	0	8 (high)
								mean	8.1

**Table 3:** Modified Newcastle-Ottawa quality assessment scale ratings for the 12 cross-sectional studies

	Representativeness of sample	Sample size	Non-respondents	Ascertainment of the risk marker	Comparability	Ascertainment of the outcome	Statistical test	Out of 10 points
CDC Medicaid. 2009	1	0	0	1	0	2	1	5 (low)
CDC Non-illicit Drugs Utah 2005	1	0	0	1	0	2	1	5 (low)
CDC Prescription opioid pain relievers. 2011	1	0	0	1	1	2	1	6 (low)
CDC Urbanization, New Mexico, 2005	1	0	0	2	2	2	1	8 (high)
Coben et al. 2010	1	0	0	1	1	2	1	6 (low)
Havens et al. 2011a	1	0	0	1	2	1	1	5 (low)
Hall et al. 2008	1	0	0	1	0	2	1	5 (low)
Hulse et al. 2001	0	0	0	1	0	2	1	4 (low)
Mack 2013	1	0	0	1	0	2	1	5 (low)
Piercefield et al. 2010	1	0	0	1	0	2	1	5 (low)
Rudd et al. 2016	1	0	0	1	1	2	1	6 (low)
Silva et al. 2013	1	0	0	0	2	1	1	5 (low)
							mean	5.4

### Characteristics of included studies

Of the 29 studies included, only three investigated study populations outside the United States (Tables 4 and 5) (Gomes et al. 2011; Hulse et al. 2001; Caudarella et al. 2016) and one exclusively examined adolescents (Hulse et al. 2001). Of the remaining 26 studies, nine considered the entire United States, including four being conducted in veterans (Bohnert et al. 2012; Bohnert et al. 2011; Seal et al. 2012; Zedler et al. 2014), and one focused on women in the general population (Mack 2013). Of the 17 studies conducted in the United States that focus on smaller geographic areas, two were located in New Mexico (Paulozzi et al. 2012; Centers for Disease Control and Prevention 2005), two were located in Utah (Centers for Disease Control Prevention 2005; Lanier et al. 2012), two were located in Washington State (Dunn et al. 2010; Centers for Disease Control Prevention 2009), two were located in West Virginia (Hall et al. 2008; Peirce et al. 2012), while the others evaluated the Appalachian counties of Kentucky (Havens et al. 2011a), Los Angeles (Silva et al. 2013), Boston (Bauer et al. 2016)), New York City (Silva et al. 2013; Cerdá et al. 2013; Brady et al. 2015), Colorado (Dilokthornsakul et al. 2016), North Carolina (Whitmire and Adams 2010), Oklahoma (Piercefield et al. 2010) and Oregon (Hartung et al. 2007). The time periods of the studies conducted in Washington State overlapped by 3 years, but each study contained data for a time period that did not overlap (Dunn et al. 2010; Centers for Disease Control Prevention 2009). Similarly, the studies in West Virginia overlap by 1 year, but one study examines all residents of the state and unintentional overdose decedents in 1 year (Hall et al. 2008), while the other evaluates residents of the state who received and filled a prescription for a controlled substance over a two and half year time period (Peirce et al. 2012). With the exception of California, Colorado, North Carolina, New York and Oregon, the majority of these locations had age-adjusted PDO death rates that were higher than the national rate (16.3 per 100,000 population) in 2015, the latest year for which data were available (Rudd 2016; Hartung et al. 2007).

**Table 4:** Risk markers and outcomes of studies evaluating prescription drug overdose

First author, year	Risk markers and exposures assessed	Outcome	Sample size
Bauer et al. 2016	Age, sex, race/ethnicity, veteran, location of death, autopsy performed	Drug overdose death	28,033
Bohnert et al. 2011 ^a^	Sex, age, race, clinical diagnoses, comorbid conditions, as well as opioid dose and schedule	Unintentional prescription opioid overdose (ICD-10 X42, X44, Y12 or Y14 in combination with T40.2)	155,434
Bohnert et al. 2012 ^a^	Sex, age, Charlson comorbidities, psychiatric diagnoses, substance use disorders, alcohol use disorders, other specific drug use or mental health disorders	Death by accidental medication overdose was an accidental death with an underlying cause of death coded as ICD-10 codes X40-X45 due in part or whole to prescription or over the counter medications (ICD-10 codes T36.0-T39.9, T40.2-T40.4, and T42.0-T50.9)	3,291,891
Brady et al. 2015	ED utilization, age, sex, race, clinical characteristics	Prescription drug overdose death	5464
Caudarella et al. 2016	Age, gender, ethnicity, homelessness, incarceration, daily cocaine injection, daily heroin injection, daily crack smoking, methadone maintenance treatment, HIV serostatus, and HCV serostatus	Overdose mortality	2317
CDC Medicaid. 2009	Sex, age, Medicaid	A Washington state resident whose death certificate had a manner of death listed as “accidental” or “natural” and one or more contributing causes coded as ICD-10 (T40.0-T40.6 and F11) and specific words compatible with acute drug intoxication recorded in any death field and a prescription opioid in any of the cause of death fields	6,321,950
CDC Non-illicit Drugs Utah 2005 ^b^	Sex, age, area of residence	Non-illicit drug poisoning death	2,281,235
CDC Prescription opioid pain relievers. 2011	Sex, age, race	Prescription drug overdose deaths (with underlying causes of deaths listed as ICD-10 codes X40-X44, X60-X64, X85 or Y10-Y14 and having T36-T39, T40.2-T40.4, T41-T43.5, and T43.7-T50.8) as contributing causes	304,093,966
CDC Urbanization, New Mexico, 2005	Urbanization	Unintentional poisoning deaths from prescription drugs (i.e. methadone, other opioid painkiller, tranquillizer/muscle relaxant, antidepressant, barbiturate, or other prescription drug)	17,919,059
Cerdá et al. 2013	Sex, age, race	Analgesic overdose fatalities (ICD-10 X40-X44, T40.0-T40.2)	3883
Coben et al. 2010	Sex and age	Hospitalizations for poisoning by prescription opioids, sedatives and tranquilizers (ICD-9965.02, 965.09, 965.5, 965.8, 967.0, 969.4, 969.5, 967.8, and 967.9.) Poisonings were classifıed as unintentional if there was an E-code present in the E850–E858 range (accidental poisonings by drugs, medicinal substances, and biologicals)	39,450,216
Dilokthornsakul et al. 2016	Sex, age, mean morphine dose equivalents, methadone use, chronic opioid use, pain diagnosis, comorbidities, history of other medication use	≥1 medical claim for an emergency department visit or a hospitalization associated with an opioid overdose	3264
Dunn et al. 2010	Sex, age, history of depression, history of substance abuse, opioid dose, any opioid use	Opioid-related overdose death, or non-fatal event defined as definite or probable opioid-related overdose	9940
Gomes et al. 2011 ^c^	No. of pharmacies dispensing opioids, daily dose of opioids (>200 mg MME)	Opioid-related death	2212
Hall et al. 2008	Sex, age, marital status and highest education	Unintentional drug overdose deaths that involved prescription pharmaceuticals. This excluded those overdoses due solely to illicit drugs, over-the-counter products, or alcohol.	182,170
Hartung et al. 2007	Type of long acting opioid (Methadone, Oxycodone, Fentanyl, Morphine)	Administrative claims for an opioid-related serious adverse event, ED encounter or hospitalization for opioid-related adverse event (CPT code 99281-99285 or 99,288 or ED revenue center codes of 45× or 981 with ICD-9965.0×), opioid poisoning (ICD-9965.0×), or overdose symptoms (ICD-9 codes 780.0×, 78.07×, 418.81, 518.82, 564.0×)	5684
Havens et al. 2011a	Sex, age, race, psychiatric comorbidities, substance use	Non-fatal overdose	400
Hulse et al. 2001	Sex	Hospitalization for prescription /over the counter drugs	160
Lanier et al. 2012	Sex, age, race, marital status, body mass index, uninsured, education, employment status, smoking status, residence in an urban county, military service	Death from prescription opioids	1562
Mack 2013	Age and race	Prescription drug overdose deaths (with underlying causes of deaths listed as ICD-10 codes X40-X44, X60-X64, X85 or Y10-Y14 and having T36-T39, T40.2-T40.4, T41-T43.5, and T43.8-T50.8) as contributing causes	157,237,928
Paulozzi et al. 2012	Sex, age, prescription history	Death from unintentional drug overdose	6293
Peirce et al. 2012	Prior doctor and or pharmacy shopping	Controlled substance-related death	1,049,903
Piercefield et al. 2010 ^d^	Sex, age, race and urban/rural	Medication overdose deaths	3,540,517
Rudd et al. 2016	Sex, age, race	Prescription drug overdose deaths (with underlying causes of deaths listed as ICD-10 codes X40-X44, X60-X64, X85 or Y10-Y14 and having T36-T39, T40.2-T40.4, T41-T43.5, and T43.7-T50.8) as contributing causes	641,538,924
Seal et al. 2012 ^a^	Opioid use, post-traumatic stress disorder (PTSD), PTSD with or without other mental health disorders	Opioid-related accidents and overdoses (ICD-9 codes: 965.00, 965.01, 965.02,965.09, E850.0, E850.1, E850.2, E935.0, E935.1, and E935.2)	141,030
Silva et al. 2013	Sex, race, psychiatric care, substance use	Non-fatal overdose on prescription opioids and/or tranquilizers	596
Turner and Liang 2015	Sex, age, US region, clinical conditions, mental health and substance use disorders,	Any drug overdose event	206,869
Whitmire and Adams 2010 ^d^	Eligibility category, race, residence, specific disorders, drug claims	Unintentional overdose death (ICD-10 X40-X49)	2801
Zedler et al. 2014	Age, sex, race/ethnicity, marital status, BMI, US Census region, comorbidities, opioid use, all-cause health care utilization (ED visits)	Occurrence of serious opioid-related toxicity or overdose as defined by listed ICD-9-CM and CPT codes	8987

*MMEs* morphine milligram equivalents, *CPT* current procedural terminology, *ICD-9* international classification of diseases, 9th revision, *ICD-10* international classification of diseases, 10th revision, *CS* controlled substances, *OME* office of the medical examiner

^a^Studies are not independent

^b^Information refers to deaths occurring between 1999 and 2003

^c^Information refers to deaths occurring between 2004 and 2006

^d^Matching variables not included in risk markers

**Table 5:** Characteristics of studies evaluating risk markers for prescription drug overdose

First author, Year	Study subjects	Data source	Study design	Location	Study time period	Source of outcome information	Source of risk marker information
Bauer et al. 2016	Adults seen at Boston Health Care for the Homeless Program (BHCHP)	BHCHP data and Massachusetts Department of Public Health annual death occurrence files	Retrospective record review (cohort)	Boston, Massachusetts	January 1, 2003 - December 31, 2008	Massachusetts Department of Public Health annual death occurrence files	BHCHP electronic health records
Bohnert et al. 2011 ^a^	All unintentional prescription opioid overdose decedents and a random sample of patients a month those individuals who used Veteran’s Health Services in 2004 or 2005 and received opioid therapy for pain	Veteran Affairs National Patient Care Database and National Death Index	Case-cohort study	United States	Fiscal Year (FY) 2004 -FY 2008	National Death Index	Veteran Affairs National Patient Care Database
Bohnert et al. 2012 ^a^	All treated in Veterans Health Administration facilities during the fiscal year 1999 who were alive at the start of fiscal year 2000.	Veteran Affairs National Patient Care Database and National Death Index	Cohort	United States	Oct 1, 1999 - Sep 30, 2006	National Death Index	Veteran Affairs National Patient Care Database
Brady et al. 2015	New York State ED patients who subsequently died of prescription drug overdose	New York Statewide Planning and Research Cooperative	Nested case-control	New York, United States	2006-2010	New York City vital statistics records	New York Statewide Planning and Research Cooperative System (SPARCS) ED data
Caudarella et al. 2016	Persons who use drugs in Vancouver, Canada - 2 cohorts: Vancouver Injection Drug Users Study (VIDUS) and AIDS Care Cohort to Evaluate Access to Survival Services (ACCESS)	VIDUS, ACCESS, and provincial Vital Statistics Agency	Prospective cohort	Vancouver, Canada	May, 1996 - December, 2011	provincial Vital Statistics Agency	Self-report
CDC Medicaid 2009	Residents of Washington State	The Washington State Heath and Recovery Services Administration	Cross-sectional	Washington State, United States	2004-2007	National Center for Health Statistics and Medicaid	National Center for Health Statistics and Medicaid
CDC Non-illicit Drugs Utah 2005 ^b^	Utah residents	Centralized state medical examiner system and office of planning and budget state population estimates	Cross-sectional	Utah, United States	1991-2003	Office of the state medical examiner	Office of the state medical examiner
CDC Prescription opioid pain relievers 2011	Drug overdose deaths in the US population	National Vital Statistics System	Cross-sectional	United States	2008	National Vital Statistics System	National Vital Statistics System
CDC Urbanization, New Mexico, 2005	New Mexico residents	New Mexico Office of Medical Investigator, Office of Management and Budget, US Census data	Cross-sectional	New Mexico, United States	1994-2003	New Mexico Office of Medical Investigator	New Mexico Office of Medical Investigator, Office of Management and Budget and U.S. census
Cerdá et al. 2013	Decedents in New York City	Office of the Chief Medical Examiner of New York City	Case-control	New York City, NY, United States	2000-2006	Office of the Chief Medical Examiner of New York City	Office of the Chief Medical Examiner of New York City
Coben et al. 2010	Hospitalizations for poisonings	National Inpatient Sample from the Agency for Healthcare Research and Quality Improvement	Cross-sectional	United States	2006	Hospital discharge data	Hospital discharge data
Dilokthornsakul et al. 2016	Colorado Medicaid population, 2009-2014	Medicaid claims database	Retrospective nested case-control	Colorado, United States	July 2009 - June 2014	Colorado Medicaid claims database	Colorado Medicaid claims database
Dunn et al. 2010	Individuals receiving care from the group health cooperative and who were age 18 years or older, initiated treatment with opioids between 1997 and 2005, received 3 or more opioid analgesic prescriptions in the 90 days after initiation and received a non-cancer pain diagnosis from the prescribing physician within 2 weeks prior to the initial opioid prescription	Group Health Cooperative claims data	Retrospective cohort	Washington State, United States	January 1, 1997 -December 31, 2006	Overdose events were assessed primarily through medical record review	Group Health Cooperative data
Gomes et al. 2011 ^c^	Residents of Ontario Canada 15-64 who were eligible for public drug coverage and had received an opioid for nonmalignant pain	Ontario Public Drug Benefit Program; Ontario Cancer Registry	Population-based nested matched case-control	Ontario, Canada	Aug 1, 1997 -Dec 31, 2006	Office of the Chief Coroner of Ontario	Ontario Public Drug Benefit Program
Hall et al. 2008	Residents of West Virginia	Death certificates- Health Statistics Center of the West Virginia Department of Health and Human Resources and cross-referenced with investigations from the Chief Medical Examiner, West Virginia Board of Pharmacy and US Census data	Cross-sectional	West Virginia, United States	2006	Death certificates- Health Statistics Center of the West Virginia Department of Health and Human Resources and cross-referenced with investigations from the Chief Medical Examiner	Death certificates- Health Statistics Center of the West Virginia Department of Health and Human Resources and cross-referenced with investigations from the Chief Medical Examiner and US. Census
Hartung et al. 2007	Patients receiving at least one script for of a long acting opioid of ≥28 day supply who had ≥180 days of continuous Medicaid fee for service eligibility prior to the first dispensing	Medicaid administrative claims data	Retrospective cohort	Oregon, United States	Jan 1, 2000 - Dec 31, 2004	Medicaid administrative claims, by ICD-9 codes	Medicaid administrative claims
Havens et al. 2011a	Residents of rural Appalachian counties in Kentucky that use drugs and are over 18 and had used drugs in the past 30 days	Respondent driven sample	Cross-sectional	Any Appalachian county of rural Kentucky, United States	Nov 2008-Sep 2010	Self-report	Self-report
Hulse et al. 2001	Adolescents (12-19 years of age) presenting to the emergency department with conditions related to alcohol or drug use who had a nurse assessment in 4 metropolitan hospitals in a 4-week period, Perth, Australia	Medical record data	Cross-sectional	Perth Australia	4-week period in 2000	Medical record	Medical record
Lanier et al. 2012	Decedents 18 years or older who died from prescription opioids in Utah from October 26, 2008 to October 25, 2009 and Utah 2008 Behavioral risk marker Surveillance System respondents who reported prescription opioids use during the previous year	Utah 2008 Behavioral Risk Factor Surveillance System	Matched case-control	Utah, United States	2008-2009	Utah Office of the Medical Examiner (OME)	OME, Next-of-kin interviews, Utah 2008 Behavioral Risk Factor Surveillance System
Mack 2013	Drug overdose deaths among women and the US population	National Vital Statistics System	Cross-sectional	United States	2010	National Vital Statistics System	National Vital Statistics System
Paulozzi et al. 2012	Decedents 10 years and older who died of unintentional drug overdose and had at least one record in the New Mexico Prescription Drug Monitoring Program and controls were identified through the state prescription drug monitoring program	New Mexico Office of Medical Investigator, and New Mexico Prescription Drug Monitoring Program data	Matched case-control	New Mexico, United States	Oct 1, 2006 - Mar 31, 2008	New Mexico Office of the Medical Investigator	New Mexico State Prescription Drug Monitoring Program
Peirce et al. 2012	Persons 18 years and older who had at least 1 outpatient prescription filled for a Schedule II through Schedule IV controlled substance in West Virginia	West Virginia State Board of Pharmacy Controlled Substance Monitoring Program and Forensic Drug Database	Case-control	West Virginia, United States	Jul 1, 2005 - Dec 31, 2007	Forensic Drug Database	West Virginia State Board of Pharmacy Controlled Substance Monitoring Program and Forensic Drug Database
Piercefield et al. 2010 ^c^	Oklahoma residence in which the medical examiner reasoned that at least one prescription or over the counter drug contributed to the unintentional drug death	Oklahoma medical examiner data and the US census	Cross-sectional	Oklahoma, United States	Jan 1, 2004 – Dec 31, 2006	Oklahoma State the Medical Examiner case record	Oklahoma OME case record
Rudd et al. 2016	Drug overdose deaths in the US population	National Vital Statistics System	Cross-sectional	United States	2013-2014	National Vital Statistics System	National Vital Statistics System
Seal et al. 2012 ^a^	Iraq and Afghanistan veterans who received at least 1 non-cancer related pain diagnosis within 1 year of entering the Veteran’s Affairs health care system	Veteran Affairs National Patient Care Database	Retrospective cohort	United States	Oct 1, 2005 - Dec 31, 2010	Veteran Affairs National Patient Care Database	Veteran Affairs National Patient Care Database
Silva et al. 2013	A sample of young nonmedical users of prescription drugs	Chain referral sampling and recruitment from different project phases	Cross-sectional	New York, New York and Los Angeles, California, United States	Oct 1, 2009 –Mar 31 2011	Self-report	Self-report
Turner and Liang 2015	Aetna Health Maintenance Program beneficiaries aged 18-64 years, enrolled at least 1 year, who filled at least two prescriptions for Schedule I or II opioids for non-cancer pain	HMO enrollment files and claims for services and prescriptions	Retrospective cohort	United States	January 2009- July 2012	HMO enrollment files and claims for services and prescriptions	HMO enrollment files and claims for services and prescriptions
Whitmire and Adams 2010 ^d^	North Carolina Medicaid population, 2007	Medicaid administrative claims data	Matched case-control	North Carolina, United States	2006-2007	North Carolina resident death records,	Medicaid administrative claims
Zedler et al. 2014	Veterans Health Administration patients who were dispensed an opioid by VHA	VHA Medical SAS datasets	Retrospective nested case-control	United States	October 1, 2010 - September 30, 2012	VHA Medical SAS Datasets	VHA Medical SAS Datasets

*MMEs* morphine milligram equivalents, *CPT* current procedural terminology, *ICD-9* international classification of diseases, 9th revision, *ICD-10* international classification of diseases, 10th revision, *CS* controlled substances, *OME* office of the medical examine

^a^Studies are not independent

^b^Information refers to deaths occurring between 1999 and 2003

^c^Information refers to deaths occurring between 2004 and 2006

^d^Matching variables not included in risk markers

The outcomes evaluated in the 29 studies included morbidity and mortality measures. Drugs assessed in these studies were not specifically limited to prescription opioids. Ten of these studies focused on prescription opioid overdose (Bohnert et al. 2011; Dunn et al. 2010; Lanier et al. 2012; Gomes et al. 2011; Seal et al. 2012; Cerdá et al. 2013; Brady et al. 2015; Dilokthornsakul et al. 2016; Hartung et al. 2007), eight examined PDO (Centers for Disease Control and Prevention 2011; Coben et al. 2010; Silva et al. 2013; Mack 2013; Centers for Disease Control and Prevention 2005; Centers for Disease Control Prevention 2009; Peirce et al. 2012; Paulozzi et al. 2011), three examined overdose from both prescription and over-the-counter (OTC) medications (Bohnert et al. 2012; Hulse et al. 2001; Piercefield et al. 2010), one examined non-illicit drug overdose (Centers for Disease Control Prevention 2005) including prescription drugs, OTC medications, and alcohol, and the remaining eight examined unintentional overdose deaths—with some indication of prescription drug use (Havens et al. 2011a; Paulozzi et al. 2012; Caudarella et al. 2016; Zedler et al. 2014; Hall et al. 2008; Bauer et al. 2016; Whitmire and Adams 2010; Turner and Liang 2015). Of the 29 studies, ten (Coben et al. 2010; Dunn et al. 2010; Havens et al. 2011a; Silva et al. 2013; Hulse et al. 2001; Seal et al. 2012; Zedler et al. 2014; Dilokthornsakul et al. 2016; Hartung et al. 2007; Turner and Liang 2015) examined nonfatal overdose. One study examined all fatal poisonings and all intents, including suicide and homicide (Rudd et al. 2016).

### Identification of risk markers

Risk marker information was extracted and evaluated for the most commonly examined variables across the 29 eligible studies: sex, age, white race, psychiatric disorders, SUDs, and urban/rural residence. Specifically, 21 studies examined sex, 13 studies examined age as a categorical variable, 14 studies examined race, 11 studies examined psychiatric disorders, 10 studies examined SUDs and five studies examined urban/rural residence.

### Synthesis and findings of the quantitative review

#### Sex

**Fig. 2:**
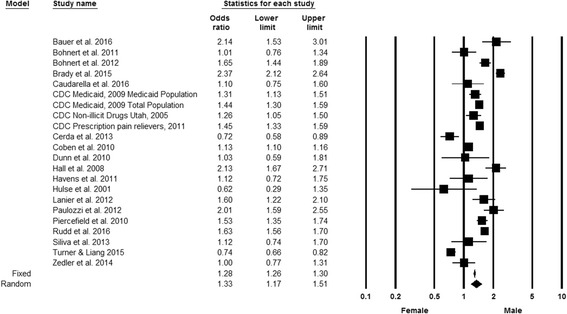
Forest plot, summary odds ratio and 95% confidence of prescription drug overdose with sex. The size of each square is proportional to the relative weight that each study contributed to the summary odds ratio. The summary odds ratio is indicated by the diamond. *Horizontal bars* indicate the 95% confidence interval. Heterogeneity: Q statistic: 553.2, df = 21, *P* < 0.0001. I^2^ = 96.2. Footnote: The Bohnert et al. (2011) and Bohnert et al. (2012) papers arose from the same underlying population. In the CDC Medicaid 2009 study, the Medicaid population is a subgroup of the total population. Note: The standard errors for the CDC Prescription opioid pain relievers 2011 and Rudd et al. (2016) studies may be smaller than what was used to calculate the confidence intervals. The sample size for this study exceeded the maximum allowed by Comprehensive Meta-Analysis software, so one digit was removed from each component of the odds ratio

Most studies found that the proportion of males who overdosed was greater than the proportion of females who overdosed. However, there was some variation in effect estimates by sex across studies. Twelve studies showed that males were at statistically significant increased risk for PDO (Centers for Disease Control and Prevention 2011; Coben et al. 2010; Centers for Disease Control Prevention 2005; Bohnert et al. 2012; Paulozzi et al. 2012; Lanier et al. 2012; Centers for Disease Control Prevention 2009; Hall et al. 2008; Bauer et al. 2016; Brady et al. 2015; Piercefield et al. 2010; Rudd et al. 2016), six studies showed that there were no statistically significant differences between sexes (Bohnert et al. 2011; Dunn et al. 2010; Havens et al. 2011a; Silva et al. 2013; Caudarella et al. 2016; Zedler et al. 2014) and three studies showed that females were at increased risk for PDO (Hulse et al. 2001; Cerdá et al. 2013; Turner and Liang 2015). Overall, the random effects model showed a statistically significant increased risk for males as compared to females (summary odds ratio (SOR) = 1.33, 95% confidence interval (CI) 1.17, 1.51) (Fig. 2). The results of the “one study removed” analysis indicated that the SOR was robust, as SOR from random effects models ranged from 1.29 to 1.38. Rosenthal’s classic fail safe N, the number of new, unpublished, or null studies that would be needed to make the overall finding not significant, was 2313 (Persaud 1996).

#### Age

**Fig. 3:**
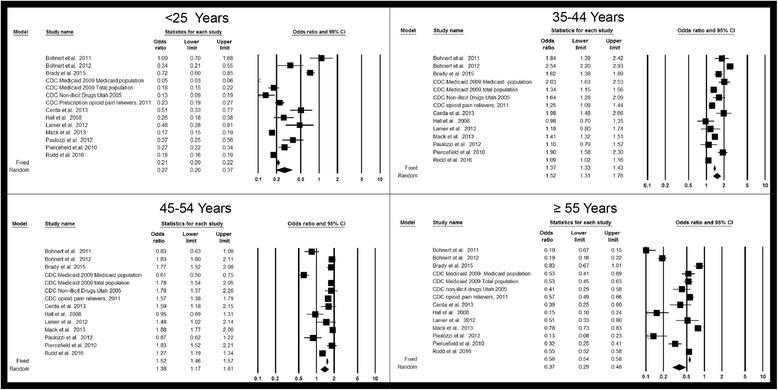
Forest plot, summary odds ratio and 95% confidence of prescription drug overdose with age. For all plots, 25-34 years is the reference group. The size of each square is proportional to the relative weight that each study contributed to the summary odds ratio. The summary odds ratio is indicated by the diamond. Horizontal bars indicate the 95% confidence interval. <25 years vs. 25-34 years Heterogeneity: Q statistic: 407.8, df = 13, *P* < 0.0001. I^2^ = 96.8; 35-44 years vs. 25-34 years Heterogeneity: Q statistic: 172.0, df = 13, *P* < 0.0001. I^2^ = 92.4; 45-54 years vs. 25-34 years Heterogeneity: Q statistic: 213.0, df = 13, *P* < 0.0001. I^2^ = 93.9; ≥55 years vs. 25-34 years Heterogeneity: Q statistic: 440.0, df = 13, *P* < 0.0001. I^2^ = 97.0. Footnote: The standard errors for the CDC Prescription opioid pain relievers 2011, Mack (2013) and Rudd et al. (2016) studies may be smaller than what was used to calculate the confidence intervals. The sample size for this study exceeded the maximum allowed by Comprehensive Meta-Analysis software, so one digit was removed from each component of the odds ratio

**Fig. 4:**
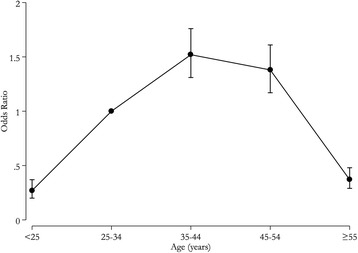
Line graph of summary odds ratios of prescription drug overdose associated with age. Error bars indicate the 95% confidence interval. <25 years vs. 25-34 years

The variation in the effect estimates across studies comparing <25, 35-44, 45-54, and ≥55 years to 25-34 years (reference group) showed that the 35-44 and 44-54 year age groups were most often at the greatest risk for overdose (Figs. 3 and 4). All studies included in Figs. 3 and 4 examined fatal overdoses. For the study conducted in Washington State (Centers for Disease Control Prevention 2009), both the Medicaid population and the total population distribution of overdoses were examined, even though the age distribution of overdose was similar in the total population and the Medicaid population.

#### Race

**Fig. 5:**
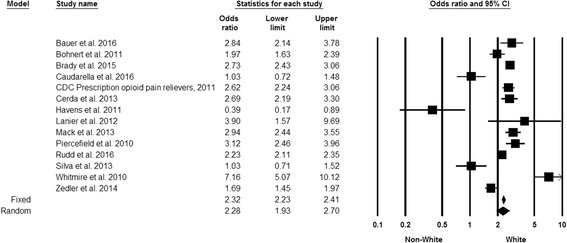
Forest plot, summary odds ratio and 95% confidence of prescription drug overdose with white race. The size of each square is proportional to the relative weight that each study contributed to the summary odds ratio. The summary odds ratio is indicated by the diamond. Horizontal bars indicate the 95% confidence interval. Heterogeneity: Q statistic: 144.2, df = 13, *P* < 0.0001. I^2^ = 91.0. Footnote: The standard errors for the CDC Prescription opioid pain relievers 2011, Mack (2013) and Rudd et al. (2016) studies may be smaller than what was used to calculate the confidence intervals. The sample sizes for these studies exceeded the maximum allowed by Comprehensive Meta-Analysis software, so one digit was removed from each component of the odds ratios

Of the 14 studies that examined race as a risk marker, 11 showed that Whites were at increased risk for PDO as compared to all other racial groups combined (Fig. 5). The overall SOR showed that Whites had statistically significant increased risk for PDO when compared to other racial groups combined (SOR 2.28, 95% CI 1.93, 2.70). Results of the “one study removed analysis” showed SOR from random effects models ranging from 2.13 to 2.42. Rosenthal’s classic fail safe N was 3785 (Persaud 1996).

#### Psychiatric disorders

In each of the 11 studies that examined psychiatric disorders, the incidence of drug overdose in individuals with psychiatric disorders was higher than in individuals without psychiatric disorders (Tables 6 and 7). However, these studies used varying definitions of conditions considered to be psychiatric disorders. The overall SOR showed that individuals with psychiatric disorders have a statistically significant increased risk for PDO compared with those who do not have psychiatric disorders (SOR = 3.94, 95% CI 3.09, 5.01). The results of the “one study removed analysis” showed SOR from random effects models ranging from 3.60 to 4.11. Rosenthal’s classic fail safe N was 7396.

**Table 6:** Risk markers for prescription drug overdose stratified by study characteristic

	Sex			Race			Psychiatric disorder			Substance use disorder			Rural residence		
	*N*	Random effects	95% CI	*N*	Random effects	95% CI	*N*	Random effects	95% CI	*N*	Random effects	95% CI	*N*	Random effects	95% CI
Unadjusted OR	22	1.33	1.17, 1.51	14	2.28	1.93, 2.70	11	3.94	3.09, 5.01	10	5.24	3.53, 7.76	5	0.93	0.72, 1.19
Outcome definition															
Fatal overdose	15	1.50	1.34, 1.68	11	2.65	2.26, 3.11	4	3.81	2.88, 5.05	4	7.05	5.15, 9.66	5	0.93	0.72,1.19
Fatal and nonfatal overdose	7	0.97	0.77, 1.21	3	0.99	0.53, 1.88	7	4.02	2.61, 6.21	6	4.08	1.78, 9.38	-	-	-
Study Design															
Case control	6	1.34	0.87, 2.06	6	2.81	2.04, 3.85	5	3.58	3.04, 4.20	5	5.65	4.10, 7.81	2	1.08	0.81, 1.43
Cohort	5	1.24	0.77, 2.01	2	1.72	0.64, 4.65	4	5.70	3.78, 8.59	3	8.34	4.32, 16.13	-	-	-
Cross-sectional	11	1.37	1.19, 1.59	6	2.07	1.61, 2.65	2	2.54	1.88, 3.43	2	2.12	1.43, 3.14	3	0.86	0.63, 1.18
Quality Assessment Score															
High (7-10)	10	1.27	0.89, 1.80	7	2.43	1.80, 3.27	9	4.29	3.32, 5.56	8	6.59	4.41, 9.85	2	0.95	0.60, 1.52
Low (0-6)	12	1.39	1.21, 1.60	7	2.14	1.68, 2.72	2	2.54	1.88, 3.43	2	2.12	1.43, 3.14	3	0.92	0.61, 1.37
Study Outcome															
Medication overdose	8	1.40	1.18, 1.65	4	2.33	1.68, 3.22	2	3.80	2.02, 7.12	2	4.84	1.42, 16.55	3	0.86	0.63, 1.18
Prescription opioid overdose	7	1.29	0.96, 1.74	4	2.50	2.06, 3.03	5	4.07	3.50, 4.72	4	6.21	4.60, 8.38	1	0.92	0.66, 1.27
Overdose of any substance	7	1.30	0.91, 1.86	6	1.98	1.37, 2.88	4	3.70	1.89, 7.24	4	4.86	1.57, 15.05	1	1.23	0.94, 1.59
Exclusions															
Hulse et al. and Coben et al.	20	1.36	1.19, 1.57	-	-	-	-	-	-	-	-	-	-	-	-
Hulse et al., Coben et al., and Cerda et al.	19	1.41	1.23, 1.62	-	-	-	-	-	-	-	-	-	-	-	-
CDC 2009 Total Population	21	1.32	1.16, 1.51	-	-	-	-	-	-	-	-	-	-	-	-
CDC 2009 Medicaid only	21	1.33	1.17, 1.52	-	-	-	-	-	-	-	-	-	-	-	-
Havens and Silva	-	-	-	12	2.54	2.17, 2.97	-	-	-	-	-	-	-	-	-
Seal et al.	-	-	-	-	-	-	10	3.79	2.95, 4.86	-	-	-	-	-	-
Seal et al., Bohnert et al. 2011, 2012	-	-	-	-	-	-	8	3.55	2.39, 5.27	-	-	-	-	-	-
Bohnert et al. 2012	-	-	-	-	-	-	-	-	-	9	4.83	2.79, 8.36	-	-	-
Havens	-	-	-	-	-	-	-	-	-	9	5.92	3.99, 8.78	-	-	-
CDC Non-illicit Drugs Utah 2005	-	-	-	-	-	-	-	-	-	-	-	-	4	0.85	0.68, 1.07
Piercefield et al. 2010	-	-	-	-	-	-	-	-	-	-	-	-	4	1.01	0.77, 1.33

**Table 7:** Risk markers for prescription drug overdose stratified by study characteristics

	Age < 25			Age 35-44			Age 45-54			Age 55 and older		
	*N*	Random effects	95% CI	*N*	Random effects	95% CI	*N*	Random effects	95% CI	*N*	Random effects	95% CI
Unadjusted OR	14	0.27	0.20, 0.37	14	1.52	1.31, 1.76	14	1.38	1.18, 1.61	14	0.37	0.29, 0.48
Outcome definition												
Fatal overdose	-	-	-	-	-	-	-	-	-	-	-	-
Fatal and nonfatal overdose	-	-	-	-	-	-	-	-	-	-	-	-
Study Design												
Case control	5	0.60	0.43, 0.83	5	1.54	1.26, 1.88	5	1.26	0.90, 1.76	5	0.30	0.12, 0.72
Cohort	1	0.34	0.21, 0.55	1	2.54	2.20, 2.93	1	1.83	1.60, 2.11	1	0.19	0.16, 0.22
Cross-sectional	8	0.17	0.14, 0.21	8	1.41	1.22, 1.64	8	1.39	1.12, 1.72	8	0.47	0.38, 0.58
Quality Assessment Score												
High (7-10)	5	0.56	0.38, 0.81	5	1.78	1.35, 2.33	5	1.33	0.98, 1.80	5	0.24	0.11, 0.55
Low (0-6)	9	0.18	0.15, 0.23	9	1.39	1.21, 1.61	9	1.40	1.14, 1.71	9	0.47	0.39, 0.58
Study Outcome												
Medication overdose	6	0.22	0.17, 0.27	6	1.57	1.23, 1.99	6	1.67	1.47, 1.90	6	0.35	0.20, 0.61
Prescription opioid overdose	6	0.34	0.14, 0.83	6	1.64	1.40, 1.93	6	1.25	0.85, 1.83	6	0.42	0.26, 0.68
Overdose of any substance	2	0.25	0.12, 0.51	2	1.09	1.03, 1.16	2	1.09	0.76, 1.56	2	0.28	0.07, 1.11
Exclusions												
Bohnert et al. 2011 and Paulozzi et al.	12	0.23	0.17, 0.32	12	1.53	1.30, 1.79	12	1.48	1.26, 1.73	12	0.44	0.35, 0.57

#### SUDs

**Fig. 6:**
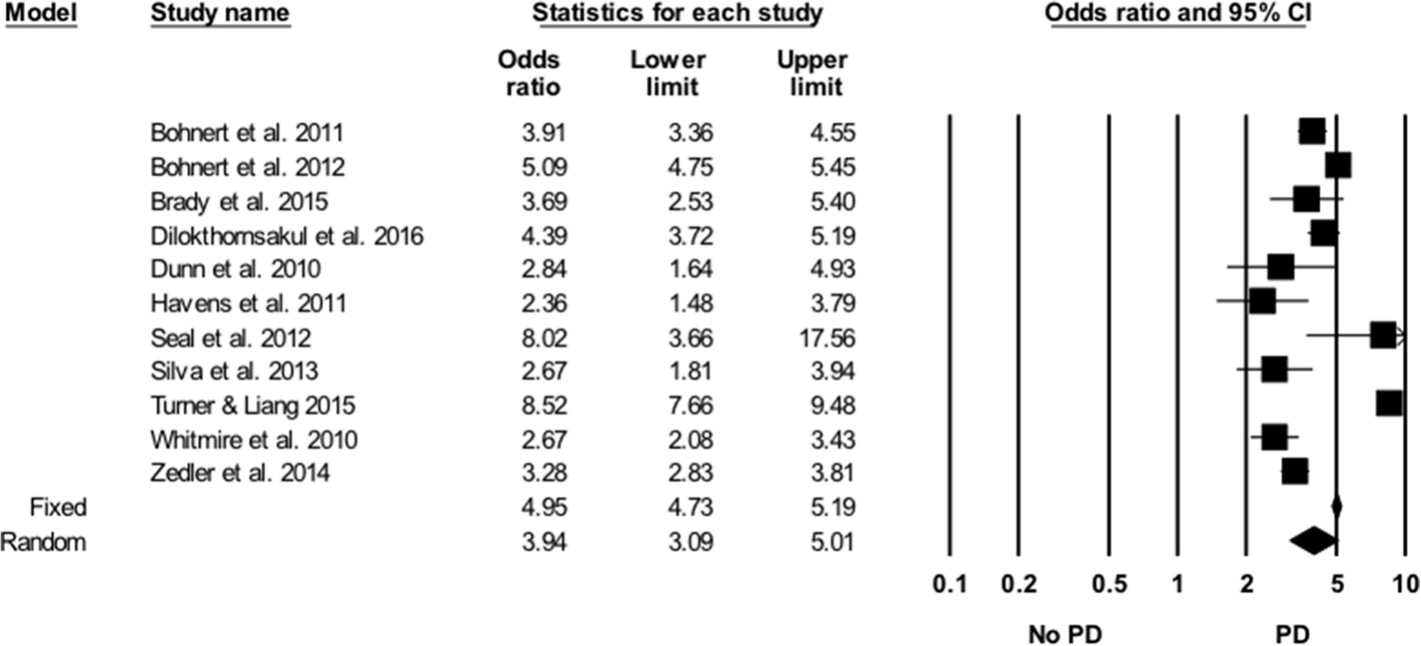
Forest plot, summary odds ratio and 95% confidence of prescription drug overdose with psychiatric disorders. The size of each square is proportional to the relative weight that each study contributed to the summary odds ratio. The summary odds ratio is indicated by the diamond. *Horizontal bars* indicate the 95% confidence interval. Heterogeneity: Q statistic: 191.2, df = 10, *P* < 0.0001. I^2^ = 94.8. Footnote: PD- Psychiatric Disorders (Definitions for each study are listed in Appendix 2). The Bohnert et al. (2011), Bohnert et al. (2012) and Seal et al. 2012 papers arose from the same underlying population

SUDs were found to be associated with an increased risk of PDO in each of the 10 studies that examined this risk marker. Although the definition of SUDs used in these studies varied, the results were generally consistent (Fig. 6). Overall, the SOR of PDO associated with SUDs was 5.24 (95% CI 3.53, 7.76). The results of the “one study removed analysis” showed that random effects SORs ranged from 4.53 to 5.92. Rosenthal’s classic fail safe N was 9465.

#### Rural residence

**Fig. 7:**
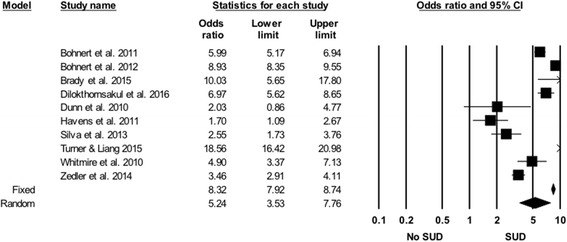
Forest plot, summary odds ratio and 95% confidence of prescription drug overdose with SUDs. The size of each square is proportional to the relative weight that each study contributed to the summary odds ratio. The summary odds ratio is indicated by the diamond. *Horizontal bars* indicate the 95% confidence interval. Heterogeneity: Q statistic: 391.9, df = 9, *P* < 0.0001. I^2^ = 97.7. Footnote: SUD – Substance Use Disorder (Definitions for each study are listed in Appendix 2). The Bohnert et al. (2011) and Bohnert et al. (2012) papers arose from the same underlying population

The five studies that examined the association rural/urban residence with the risk of PDO reported conflicting results. The estimated ORs of PDO associated with rural areas compared to urban areas varied considerably across studies (Fig. 7). The random effects SOR of PDO associated with rural residence was not statistically significant (SOR = 0.93, 95% CI 0.72, 1.19).

#### Heterogeneity

**Fig. 8:**
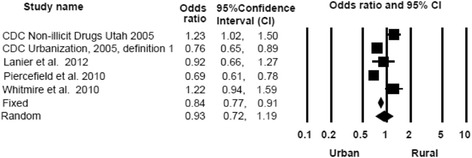
Forest plot, summary odds ratio and 95% confidence of prescription drug overdose with rural residence. The size of each square is proportional to the relative weight that each study contributed to the summary odds ratio. The summary odds ratio is indicated by the diamond. *Horizontal bars* indicate the 95% confidence interval. Heterogeneity: Q statistic: 34.5 *P* < 0.0001. I^2^ = 88.4. Footnote: Definition 1: Counties in metropolitan areas are considered urban and the remaining counties of residence were categorized as rural

Heterogeneity in effect estimates was assessed with the Q and I^2^ statistics and was found to be significant for each of the six risk markers examined (Figs. 2, 3, 5, 6, 7 and 8). Heterogeneity in effect estimates persisted across each of the six risk markers examined when stratifying by study design, quality assessment score, type of substances used, and whether the study examined fatal overdose or fatal and non-fatal overdose (not shown).

## Discussion

Given the current drug overdose crisis in the United States, it is important to understand risk markers for PDO and understand how findings may vary across studies. This meta-analysis summarizes published literature on PDO for six risk markers that were most frequently assessed in the literature: male sex, age 35-44 years, white race, comorbid psychiatric disorder diagnosis, comorbid diagnosis of a SUD and urban/rural area of residence. While, none of the risk markers identified in this systematic review are easily modifiable, understanding them may help identify individuals at heightened risk for PDO.

While males are at greater risk for PDO on average, sex is a relatively weak and inconsistent risk marker – resulting in an overall 20% increase in PDO in men. Six studies did not find any increase in risk for PDO for males as compared to females (Bohnert et al. 2011; Dunn et al. 2010; Havens et al. 2011a; Silva et al. 2013; Caudarella et al. 2016; Zedler et al. 2014). Three studies found the females were at increased risk for PDO, which may be a consequence of the study populations examined in these two studies (Hulse et al. 2001; Cerdá et al. 2013; Turner and Liang 2015). First, Cerdá et al. (2013) found that in comparison to females, males in New York City appear to be at greater risk for other accidental death relative to opioid analgesic PDO. The study’s finding of increased risk for unintentional opioid analgesic PDO in women in New York City may be related to the authors’ choice of reference group (non-overdose-related fatal accidents). It is known that overall in the United States males are at greater risk for accidental death than women (Waldron et al. 2005). Thus, men in the reference group have a lower risk of fatal overdose than the general population as well as those experiencing non-accidental death, resulting in higher estimates of gender differences (Sorenson 2011; Insurance Institute for Highway Safety 2017). Second, Hulse et al. (2001) studied a small group of adolescents seeking treatment in the ED for alcohol or drug related ailments and found females have a greater risk for non-fatal prescription drug related overdose in comparison to males. The Hulse et al. finding is consistent with literature on rates of hospitalization and ED visits for prescription drug use and non-fatal poisoning which show women are more likely than men to utilize care for prescription drug misuse or non-fatal poisoning (Cai et al. 2010; Unick et al. 2013; Xiang et al. 2012). Finally, Turner et al. studied HMO beneficiaries and note that they may miss deaths occurring out of network or outpatient deaths. Women in the United States are more health-seeking than men and hospitalization for PDO is higher for women than for men, which may explain the increased risk for PDO in women compared to men observed in this study (Coben et al. 2010; Turner and Liang 2015).

Understanding the relationship between age and PDO is complicated by the fact that many studies examine different age categories. Increased risk for overdose is highest in the 35-44 age group, which in part may result from a cohort effect arising from the aging baby-boomer generation (Miech et al. 2011). However, this review did not specifically assess birth cohort as a risk marker. Additionally, age and physical condition affect one’s ability to metabolize drugs (Smith 2009). Several studies have posited on the negative long-term implications of nonmedical use of prescription drugs in adolescents and young adults, including greater risk for dependence and bearing children dependent on prescription drugs (Whiteside et al. 2013; Patrick et al. 2012; Compton and Volkow 2006a, 2006b).

Whites have higher fatal and non-fatal PDO rates than other races. While the other studies included in this meta-analysis evaluated risk for fatal PDO, the only study that did not find that Whites were at increased risk for overdose evaluated self-reported nonfatal overdose incidents (Havens et al. 2011a). This study of nonfatal overdose incidents (Havens et al. 2011a) also used respondent-driven sampling – a method for sampling hard-to-reach populations – which may have resulted in a sample that is more homogeneous than would be obtained with traditional sampling methods (Havens et al. 2011a; Heckathorn 1997). Other studies have found that White race is associated with an increased risk for alcohol dependence, and heroin (Hasin et al. 2007; Woerle et al. 2007; Calcaterra et al. 2013). There are other reasons why non-Whites may be at lower risk for overdose related to differences in treatment for pain in White and non-White patients. It has been found that Black patients are less likely to be assessed and treated for pain than White patients (Hoffman et al. 2016). In survey respondents, this under-treatment for pain in Black patients has been linked to inaccurate beliefs among medical students and residents that Blacks patients experience less pain than White patients due to false assumptions about biological differences (Hoffman et al. 2016). Even when assessed for pain and being prescribed pain medication, surveys of pharmacies in New York City indicate that pharmacies in non-White neighborhoods were less likely to stock prescription opioid medications than pharmacies in White neighborhoods (Green et al. 2005; Singhal et al. 2016; Morrison et al. 2000). Decreased access to and availability of prescription opioids to non-White patients in comparison to White patients may translate to less prescription opioid use and less risk of PDO.

Across studies, this review found that patients with psychiatric disorders were at increased risk of PDO. There are many reasons that patients with psychiatric disorders are at increased risk for PDO. First, patients with psychiatric disorders are often prescribed medication to treat their illness and these medications, (e.g. benzodiazepines, anti-depressants, barbiturates) interact with opioids to increase the risk of fatal and non-fatal overdose (Jones et al. 2012; Maurer and Bartkowski 1993; U.S. Food and Drug Administration 2016). Patients with psychiatric disorder may also self-medicate with alcohol, which combined with opioids, can also increase the risk of fatal or non-fatal overdose (Gudin et al. 2013). Additionally, psychiatric and substance use disorders often co-occur (Mueser et al. 1998; Nunes and Levin 2004). While all studies examined found increased risk for PDO, there was significant heterogeneity in the effect estimates across studies. The heterogeneity in the psychiatric disorder meta-analysis results may stem from the fact that each of these risk markers is a general categorization that groups multiple psychiatric disorders when in fact, certain psychiatric disorders, such as anxiety disorders or depressive disorders, may have greater risk for PDO than other psychiatric disorders (Seal et al. 2012). Further, the prevalence of specific psychiatric disorders may differ from study to study, leading to varied effect estimates. Similarly, the prevalence of risk for specific psychiatric disorders among these populations e.g. populations that experience more stressors, such as veterans, may be at greater risk for PDO than the general population, may contribute to heterogeneity. Additionally, heterogeneity in the study ORs may result from lack of specificity in the study outcome. One study, Seal et al. (2012), examines the effect of having any psychiatric disorder inclusive of SUDs, which likely results in a greater odds ratio when compared with the other studies which do not include SUDs in their definition of psychiatric disorders. Seal et al. also examines prescription opioid-related accidents. Many more people suffer from opioid-related adverse events (e.g. constipation, malaise, fatigue, lethargy, respiratory failure, non-fatal opioid poisoning) and opioid-related accidents than die from prescription opioid-related intoxication (Seal et al. 2012; Hartung et al. 2007). Additionally, Seal et al. restrict their analysis to veterans experiencing serious non-cancer pain and thus, the findings of this study might not be generalizable to veterans experiencing cancer-related pain or non-veterans (Bohnert et al. 2011; Seal et al. 2012). Bohnert et al. (2011) found that risk for opioid overdose death was greater for veterans with cancer-related pain than for veterans with non-cancer related pain. These possible explanations highlight the fact that crude study odds ratios included in the meta-analysis may differ in the degree to which they are affected by confounding and effect measure modification.

The heterogeneity in the SUDs finding likely stems from the different definitions of a SUD in these studies. The two studies with lower odds ratios used atypical definitions of SUDs (Dunn et al. 2010; Havens et al. 2011a). In Dunn et al. (2010), the definition of SUD is “substance abuse”. In the 2013, Diagnostic and Statistical Manual of Mental Health Disorders, the diagnostic terms “substance abuse” and “substance dependence” were replaced in favor of “SUD.” It may be that individuals categorized as having “substance abuse” had less risk of PDO than people who would be categorized as having an “SUD.” The Havens et al. (2011a) study is cross-sectional and examined non-fatal overdose in rural drug users. It did not examine SUDs per se, but rather “ever going to drug treatment” (Havens et al. 2011a). Given that this study examined a rural area and that there is a lack of basic substance abuse treatment services in rural areas and drug treatment services are underutilized in rural areas, it is likely that drug treatment utilization would not be prevalent. Operationalizing “Ever going to drug treatment” as a diagnosis of an “SUD” may be a poor proxy in a rural setting.

The findings regarding urban/rural as a risk marker are mixed. The largest increases in PDO death rates have occurred in rural areas with rates of PDO deaths in rural areas reaching and sometimes exceeding those in urban areas (Paulozzi and Xi 2008; Park and Bloch 2016). Rossen et al. (2013) found that drug poisoning death rates in rural areas in the United States increased nearly 400% from 1999 to 2009, while death rates in urban areas in the United States increased almost 280% from 1999 to 2009. Drug poisoning trends increased more steeply in rural areas because the age-adjusted poisoning death rates were much lower in rural areas at the start of the time period (Rossen et al. 2013). Notably, the highest drug poisoning death rates were found in central metropolitan areas (Rossen et al. 2013). Thus, there is an interaction between urban/rural location and time. An alternative explanation for the heterogeneity in the effect estimates of urban/rural status on PDO may stem from the wide variation in the definitions of “urban” and “rural” used across studies. There is no one accepted definition of “urban” and “rural”. Some studies used micropolitan and metropolitan areas to define urban (Centers for Disease Control Prevention 2005; Centers for Disease Control and Prevention 2005); others identified “urban” and “rural” counties according to varying thresholds of population density (Lanier et al. 2012; Piercefield et al. 2010), and one study used county accountability regions (Appendix 2) (Whitmire and Adams 2010). These varying definitions may mean that areas of varying urbanicity and rurality are being grouped together and compared, which would result in an attenuation of the effect of urban/rural on PDO.

Several studies have reported that the epidemics of PDO and nonmedical prescription drug use have not been concentrated in metropolitan areas (Wunsch et al. 2009; Paulozzi and Xi 2008; Centers for Disease Control and Prevention 2005; Havens et al. 2011b; Wang et al. 2013). This is noteworthy because previous research on drug use and drug overdose epidemics has focused on urban areas (Coffin et al. 2003; Hembree et al. 2005). Research aimed at understanding differences in urban/rural prescription drug use is scant. To help appreciate why there are urban/rural differences in nonmedical opioid use, Keyes et al. (2014) conceptualized several reasons why individuals residing in rural counties may be vulnerable to nonmedical prescription drug use and abuse. Keyes et al. hypothesized that increased sales of opioids in rural areas led to greater availability for nonmedical use of prescription opioids and that close-knit kinship and social networks allowed for faster dispersion of prescription opioids for those at risk. Further, Keyes et al. (2014) posit that increasing economic hardship and unemployment and out-migration of upwardly mobile young adults create environmental stressors that contribute to risk for drug abuse. Cicero and colleagues (2007) found that in areas where there was more therapeutic use of prescription opioids there was also more prescription opioid abuse. Wang and colleagues examined factors associated with nonmedical prescription opioid use, and found that correlates of nonmedical prescription opioid use were similar for urban and rural areas (Higgins et al. 2003). Other geographical constructs, such as region or state, may interact with the urban/rural context. Regional and local prescribing patterns and availability of other drugs may affect PDO risk.

### Strengths and limitations

This systematic review uses standard meta-analysis guidelines to quantitatively assess risk markers for PDO. The findings may aid clinicians in identifying patients at heightened risk for PDO. This review is also comprehensive because it covers studies conducted over several decades and in different population groups.

Results from this meta-analysis should be interpreted with caution. First, the studies reviewed did not focus exclusively on PDO or PDO death. This review includes studies that examined non-fatal overdose (Coben et al. 2010; Havens et al. 2011a); PDO as defined in this study may be related to combining prescription and illicit drug use (Paulozzi et al. 2012) and may include PDO of intentional and underdetermined intent (Rudd et al. 2016). The inclusion of these studies decreases the specificity of the outcome measurement. However, non-fatal overdose is understudied. Examining non-fatal overdose is important because these incidents are near misses and can help researchers understand more about the rarer occurrence of overdose death. Further, it is not always clear-cut what should be categorized as PDO and if it should be separated from overdose overall.

Second, this review presents SOR from the random effects model in the presence of unexplained heterogeneity for each of the six risk markers. Some researchers favor not presenting a SOR in the presence of a large amount of unexplained heterogeneity since summary measures generated from random effects models are not always more conservative than summary measures of fixed effect estimates (Higgins et al. 2003; Higgins and Green 2011). Additionally, large unexplained heterogeneity suggests that studies may be evaluating different effects or compounding biases (Higgins et al. 2003; Higgins and Green 2011). While unexplained heterogeneity is not uncommon in meta-analyses of observational studies, it means that less emphasis should be placed on the SOR. Further, this heterogeneity may stem for factors that were not able to be controlled for in this study, such as changing in tolerance to opioids or mixing of different drugs including prescription opioids, heroin or illegal manufactured fentanyl.

Third, this meta-analysis did not include several important risk markers because the number of studies examining these risk markers was too small. Paulozzi et al. (2012), Lanier et al. (2012) and Hartung et al. (2007) reported that type of opioid was strongly associated with PDO death. Type of drug, dose, potency of opioid and duration action, and polysubstance use are all related to risk of PDO (Paulozzi et al. 2012; Peirce et al. 2012; Volkow and Thomas 2016). Additionally, frequent ED utilization and doctor shopping are found to be extremely predictive of subsequent PDO death and their associations with PDO are much stronger than with the risk markers examined in this review (Peirce et al. 2012; Brady et al. 2015). Future systematic reviews may update the present meta-analysis when more epidemiologic evidence has accumulated for emerging risk markers, such as type of drug, dose, potency of opioid, duration of action, polysubstance use, ED utilization and other healthcare utilization.

Finally, the literature included in this review spanned a long period of time. It is likely that risk markers and the strength of their association may change over time. For instance, geospatial risk for PDO may change with drug availability and implementation of laws to prevent diversion. Additionally, during parts of the study period, changes in the availability of drugs and opioids may lead to changes in associated risk markers. Spikes in PDO deaths have been associated with long-acting opioids, such as methadone at certain times and other times, potent legally and illegally produced opioids, such as fentanyl have been related to increases in PDO deaths (Rudd 2016; Dunn et al. 2010; Paulozzi et al. 2012; Lanier et al. 2012).

## Conclusions

This meta-analysis assesses six risk markers for PDO that were commonly examined across studies of drug overdose: sex, age, race, comorbid psychiatric disorders, SUDs and area of residence. It reveals that SUD is the risk marker most strongly associated with PDO, followed by psychiatric disorders, white race, age 35-44 years and male sex. Rural residence does not appear to be significantly associated with PDO. Future research on PDO can address gaps in the literature, such as the underlying reasons for prescription drug use, and understanding the comorbidities and health utilization patterns to help identify individuals at greatest risk for PDO. Findings of this review may aid clinicians in risk assessment while prescribing opioid analgesics and inform policymakers to more effectively allocate resources for intervention programs.

## Appendix 1

### Search strategy

Medical Subject Headings (MeSH) for Medline OVID:

1. explode prescription drugs/ and explode drug overdose/;
2. explode prescription drugs/ and explode Poison Control Centers/;
3. (explode prescription drugs/ or explode Prescription Drug Misuse/ and explode Opioid-Related Disorders/ or explode Substance-Related Disorders/ or substance-related disorders/ or amphetamine-related disorders/ or drug overdose/ or neonatal abstinence syndrome/ or opioid-related disorders/ or psychoses, substance-induced/ or substance withdrawal syndrome/ or “phenomena and processes (non-MeSH)”/) and (explode Analgesics/ or explode Prescription Drug Misuse/ and explode Analgesics/ or explode Controlled Substances/).

MeSH for PsycInfo:

1. explode Prescription Drugs/ or explode Analgesic Drugs/ or explode opiates/ and explode Drug Overdoses/or explode Drug Abuse/ or explode Drug Dependency/.

The automated search used the following search strategy for indexed and non-indexed databases: (overdose or substance misuse or drug-related death or addiction disorder) or (non-fatal overdose or prescription drug misuse) or (opioid-related death or (opioids and mortality) or drug poisoning) and (chronic pain or chronic pain patients or opioid therapy or opioid analgesic or prescription pain reliever or controlled substances or prescription drug).

## Appendix 2

**Table 8:** Definitions for psychiatric disorder and substance use disorder by study

	Risk marker definition		
First author, Year	Psychiatric Disorder (PD)	Substance Use Disorder (SUD)	Rural/Urban
Bohnert et al. 2011 ^a^	Non-substance use psychiatric disorders	Any SUD including alcohol	N/A
Bohnert et al. 2012 ^a^	Bipolar I or II disorders, Any depressive disorder, Post-traumatic stress disorder, other anxiety disorder, and schizophrenia	Any SUD including alcohol	N/A
Brady et al. 2015	Depression diagnosis (ICD-10 codes 298, 311, 309.0, 309.1)	Drug dependence (ICD-10 code 304)	N/A
CDC Non-illicit Drugs Utah 2005	N/A	N/A	Davis, Weber and Salt Lake City and Utah counties were categorized as urban and the remaining counties were categories were categorized as rural
CDC Urbanization, New Mexico, 2005 ^b^	N/A	N/A	Definition 1: Counties in metropolitan areas are considered urban and the remaining counties of residence were categorized as rural Definition 2: Counties in metropolitan or micrpolitan areas are considered urban and the remaining counties of residence were categorized as rural
Dilokthornsakul et al. 2016	“Other psychiatric illness” (includes depression, bipolar/mixed mania, schizophrenia, anxiety/panic/obsessive compulsive, personality disorder, other psychosis; excludes drug/alcohol abuse)	Drug/alcohol abuse (ICD-9 codes 303.xx, 304.xx)	N/A
Dunn et al. 2010	Depression diagnosis	Substance abuse diagnosis two years prior to entry in the cohort	N/A
Havens et al. 2011a	Major depressive disorder, generalized anxiety disorder, post-traumatic stress disorder, or antisocial personality disorder	Ever in drug treatment	N/A
Lanier et al. 2012	N/A	N/A	The Utah Department of Health considers an urban county to have a population density exceeded 100 persons per sq. mile. Using this classification, only four counties in the state of Utah are considered “urban”: Salt Lake, Davis, Utah, and Weber.
Piercefield et al. 2010	N/A	N/A	County of residence was considered urban if the county population exceeded 500 persons per sq. mile of land area, with the remainder termed rural counties. Two counties: Oklahoma and Tulsa were categorized as “urban”.
Seal et al. 2012	Mental health diagnoses (ICD-CM-9 codes 290-319: including depressive disorders, anxiety disorders, alcohol use disorders, drug use disorders) and post-traumatic stress disorder	N/A	N/A
Silva et al. 2013	Care in a psychiatric hospital	Ever in drug treatment	N/A
Turner and Liang 2015	Depression diagnosis	“Other substance abuse” (excludes alcohol abuse)	N/A
Whitmire and Adams 2010	“Mental disorders” excluding drug dependence	Drug dependence	Rural/urban classification variable was created based on the North Carolina accountability regions. The ten counties that were a part of the accountability regions and used to define the “urban” classification are: Buncombe, Cumberland, Davidson, Durham, Forsyth, Gaston, Guilford, Mecklenburg, Onslow, and Wake counties.
Zedler et al. 2014	Depression diagnosis	Substance abuse and nonopioid substance dependence (excludes opioid dependence)	N/A

^a^Not independent study populations overlap by 2 years

^b^Metro and micropolitan area definitions: https://www.census.gov/geographies/reference-files/time-series/demo/metro-micro/historical-delineation-files.html

## Abbreviations

CDC: Centers for disease control and prevention
CI: Confidence interval
df: Degrees of freedom
ED: Emergency department
OTC: Over-the-counter
PDO: Prescription drug overdose
SOR: Summary odds ratios
SUD: Substance use disorder
